# Persistent Infection with Herpes Simplex Virus 1 and Alzheimer’s Disease—A Call to Study How Variability in Both Virus and Host may Impact Disease

**DOI:** 10.3390/v11100966

**Published:** 2019-10-20

**Authors:** Colleen A. Mangold, Moriah L. Szpara

**Affiliations:** Department of Biochemistry and Molecular Biology, Center for Infectious Disease Dynamics, and the Huck Institutes of the Life Sciences, Pennsylvania State University, University Park, PA 16802, USA; cav154@psu.edu

**Keywords:** herpes simplex virus 1, neuron, Alzheimer’s disease, virus strain, neuroinflammation

## Abstract

Increasing attention has focused on the contributions of persistent microbial infections with the manifestation of disease later in life, including neurodegenerative conditions such as Alzheimer’s disease (AD). Current data has shown the presence of herpes simplex virus 1 (HSV-1) in regions of the brain that are impacted by AD in elderly individuals. Additionally, neuronal infection with HSV-1 triggers the accumulation of amyloid beta deposits and hyperphosphorylated tau, and results in oxidative stress and synaptic dysfunction. All of these factors are implicated in the development of AD. These data highlight the fact that persistent viral infection is likely a contributing factor, rather than a sole cause of disease. Details of the correlations between HSV-1 infection and AD development are still just beginning to emerge. Future research should investigate the relative impacts of virus strain- and host-specific factors on the induction of neurodegenerative processes over time, using models such as infected neurons in vitro, and animal models in vivo, to begin to understand their relationship with cognitive dysfunction.

## 1. Introduction

With the advent of recent advances in healthcare, the elderly population has been growing, and is expected to more than double by the year 2050 [1]. Despite this positive trend in life expectancy, advanced age brings an increased risk for development of neurodegenerative diseases, many of which are devastating in their pathologies. Common features of normative brain aging include alterations in brain and neuron volume, dendritic complexity, neurotransmission, and the accumulation of specific neurotoxic proteins [2,3]. It is believed that the manifestation of these alterations contributes in part to the neurodegenerative processes and cognitive deficits associated with advanced age. Despite these implications, the etiology of many of the hallmark changes in the brain that occur with aging remain unclear. However, it is widely believed that the induction of low levels of inflammation, combined with decreases in the clearance of misfolded proteins, may play critical roles in neurodegeneration and cognitive decline [4,5,6].

Alzheimer’s disease (AD) and related dementias affect over 40 million people globally, and this number is expected to rise with the steady increase in the aged population [7]. AD is characterized by behavioral and psychological symptoms including, but not limited to, memory loss, agitation, anxiety, depression, and delusions [8]. There are two main types of AD, familial and sporadic. While the symptoms of disease between the two types are indistinguishable from each other, the age of onset, as well as family history, differ. Familial AD is thought to be triggered by heritable elements, and typically begins to manifest in patients before the age of 60. In contrast, the exact causes of sporadic AD are relatively complex and ill-defined, involving a number of environmental and genetic factors, with symptoms often beginning to appear after age 60 to 65 [9,10,11,12,13,14,15,16]. Increased inflammation in the brain with advanced age, combined with the accumulation of neurotoxic proteins, including amyloid beta and hyperphosphorylated tau, are believed to be key factors in the manifestation of sporadic AD [17,18].

There is a growing body of literature linking infection by specific viruses, bacteria, and fungi with the development of neurodegenerative diseases later in life, including AD, Parkinson’s disease (PD), amylotropic lateral sclerosis (ALS), and multiple sclerosis (MS) [19,20]. It has been hypothesized that an increased proinflammatory state associated with advanced age may in part be due to the immune system’s continuous battle with microbial antigens over time [4]. This chronic parainflammation would in turn contribute to the development of neurodegenerative disease [6]. Additionally, recent evidence suggests that amyloid beta, a clinical indicator of AD, may serve as an antimicrobial peptide that restricts pathogen movement and disrupts microbial membranes [21,22]. Continuous production of amyloid beta in response to persistent microbial infection over time may lead to the pathological accumulations associated with AD. Together, these data support the hypothesis that infection by different microbes may contribute to AD pathogenesis [23]. This may be especially true for persistent infections localized within neurons, such as alphaherpesviruses. Infection with herpes simplex virus 1 (HSV-1) in particular has been linked to the development of neurodegenerative disease including sporadic AD, and has also been implicated in the manifestation of cognitive deficits observed in patients with schizophrenia [19,24,25,26,27].

HSV-1 is a double-stranded DNA virus that infects over half the global population, and triggers the manifestation of recurrent painful orofacial and/or genital lesions [28]. HSV-1 infection can cause infectious keratitis, and in rare cases encephalitis [29], and can lead to dangerous neonatal infection in roughly 4000 infants annually [30]. HSV-1 actively replicates in mucosal epithelial cells, and subsequently enters innervating peripheral sensory nerve endings [31]. The virus then traffics in a retrograde direction back to neuronal cell bodies located in the peripheral ganglia. Once in the neuronal cell body, it enters a nonreplicative, latent stage, where viral DNA becomes circularized to form an episome and lytic gene expression is silenced [32,33]. The ability of HSV-1 to infect and maintain a nonreplicative state in peripheral neurons allows for lifelong infection, as the only approved therapeutics used to treat HSV-1 target actively proliferating virus. Numerous recent publications have linked HSV-1 infection and/or seropositivity with the development of sporadic AD later in life [24,26,34,35,36,37,38,39,40,41]. It is possible that latent HSV-1 infection may over time cause the accumulation of misfolded proteins and enhance local persistent parainflammation, leading to the manifestation of disease. However, at present, gaps in knowledge exist definitively linking HSV-1 infection with the development of AD pathology. It is important to note that HSV-1 strains isolated from independent patients can differ in 2–4% of the viral genome [42,43,44,45,46,47,48]. Individual strains of HSV-1 exhibit differing abilities to reach the central nervous system (CNS) when inoculated at peripheral sites in animal models, highlighting strain-specific differences in neuroinvasion [49,50]. Despite the known diversity that exists between individual circulating HSV-1 strains, the collective impacts of these viral genomic differences and host genetic predisposition(s) on disease development and severity remain unclear. Relatively few studies have sought to detail the virus strain- and/or host-specific characteristics that may potentially influence the dynamic range of HSV impacts on disease outcomes later in life [51,52,53,54,55,56,57,58,59].

**Figure 1:**
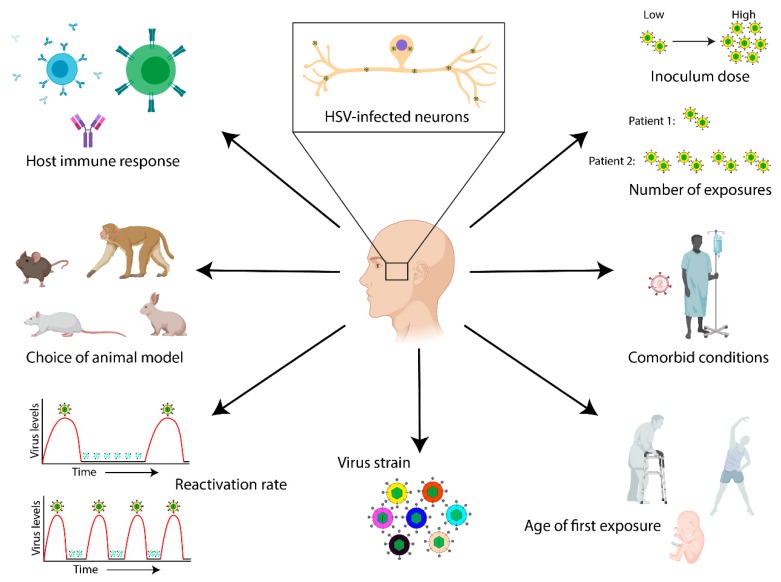
Persistent infection with herpes simplex virus 1 (HSV-1) and Alzheimer’s disease (AD): Opportunities for future research. Current evidence suggests that there is an association between HSV-1 infection and the development of AD later in life. However, a number of knowledge gaps exist in our understanding of the host- and virus-specific factors that may contribute to disease manifestation. Different elements may influence HSV-1 latency, reactivation, and disease outcomes in both natural and experimental settings. These variables should be taken into consideration when interpreting data, and in discussing the interactions between HSV-1 and AD development. These variables include (counter-clockwise from top left): Host immune responses and immune control; Choice of animal model; Reactivation rate differences between patients; Genetic differences between HSV-1 isolates or strains; Age at first exposure; Comorbid conditions (e.g., HIV infection, chronic obstructive pulmonary disease, etc.); Environmental influences, such as viral dose and number of exposures. Immune cell, animal, and host illustrations from Biorender.com.

In this review, we aim to emphasize the importance of filling these knowledge gaps, as this may help better inform physicians as to the choice and aggressiveness of AD therapy on a more individualized basis (e.g., the use of anti-herpetic medication [41]). We begin here by discussing the specific terminology used to describe viral infection phenotypes, since many of the labs working in the emerging field of microbial links to AD are not classically trained in virology. We discuss how the terms chronic, latent, and persistent are used in the literature to describe specific characteristics of HSV-1 infection. We follow this by detailing the contributions of neuroinflammation and neurotoxic protein accumulation to the development of neurodegenerative processes and disease. We then discuss the potential connections between persistent HSV-1 infection, neuroinflammation, and neurotoxic protein accumulation, as well as the hypothesized mechanisms underlying HSV-1-induced disease development. We close by emphasizing the importance of characterizing virus strain- and host-specific interactions on an individual basis, since these may influence the potential for disease development later in life in HSV-1-infected patients. When investigating these interactions, it will be important to consider how factors such as host or animal model, age of first exposure, virus strain, inoculum dosage, frequency of exposure, comorbid conditions, and other factors may influence disease outcome in both natural and experimental settings (Figure 1).

## 2. Definitions of Latent, Persistent, and Chronic Viral Infection

In order to understand the contribution of infection with a pathogen to the development of disease later in life, a clear understanding and precise description of viral infection characteristics is required. In many instances in the literature, the terms “latent”, “persistent”, and “chronic” are used interchangeably to describe the infection dynamics of different viruses. This becomes problematic when trying to frame the discussion of disease development later in life, with host-virus interactions that occur over the course of a patient’s lifetime.

### 2.1. Definitions

**Figure 2:**
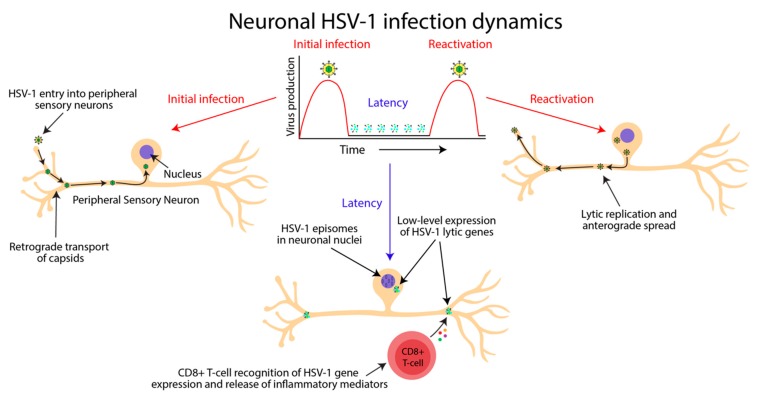
Persistent herpes simplex virus 1 (HSV-1) infection has periods of latency interspersed with periods of reactivation. The definition of latent viral infection requires a lack of infectious virus production between periods of active lytic replication. Infectious virions enter the nervous system at sensory nerve endings from the site of initial infection and are trafficked in retrograde to neuronal nuclei within peripheral ganglia. Within the neuronal nuclei, HSV-1 genomes form episomes, and lytic replication is repressed. However, during this time, low levels of lytic gene expression occur in the absence of infectious virion production, inducing local immune responses to dampen viral gene expression. Upon reactivation, infectious virions are produced and are trafficked in an anterograde direction back to the site of infection, causing recurrent skin infection and transmission to new hosts. The lack of infectious virion production and the formation of episomal DNA within host neuronal nuclei demonstrates that HSV-1 is not a chronic infection (i.e., with constant low-level virus production), despite its ability to produce low levels of lytic gene expression during latency. Diagram: neuronal cell cartoons from BioRender.com, graphical representation of HSV-1 infection dynamics adapted from [60].

Both latent and chronic infection can be defined as specific subtypes of a persistent viral infection where the virus is never cleared (Figure 2). These are unlike acute viral infections, such as influenza [60]. Instead, during persistent infections, viral genomes reside permanently within specific host cells by integrating into cellular DNA or by forming episomes [60]. In the case of HSV-1, long term persistence occurs via episomes held in the nuclei of latently infected neurons. Although quite similar in definition, latent and chronic infections have specific characteristics that set them distinctly apart. Infection with a virus that is able to establish a latent state in its host results in recurrent disease over a patient’s lifetime. In between instances of active disease, there is a lack of infectious virus production. In contrast, a chronic viral infection continuously produces infectious virus, regardless of overt disease status [60].

### 2.2. HSV-1 Causes a Persistent, Latent Infection in Neurons

The lifecycle of HSV-1 requires lytic infection of epithelial cells and quiescent infection of neurons [31]. Localization within neurons has been theorized to provide HSV-1 with an “immunologically-privileged” reservoir in which to effectively evade immune clearance [60]. However, neurons cannot be considered immune-privileged, given the diversity of immune signaling that is known to occur in the nervous system [61,62,63,64,65,66,67,68] (see Section 3.1 for further discussion). Given that infection endures over the course of a patient’s lifetime and is never cleared, HSV-1 infection is defined as persistent. The current paradigm holds that while HSV-1 is able to actively replicate in mucosal epithelial cells, lytic gene expression is repressed in neurons [31]. This occurs as a consequence of a number of molecular events, including epigenetic modification, changes in host cell signaling, and heterochromatin formation [31,33,69,70]. However, low levels of lytic viral gene transcription may still occur in latently-infected neurons. Historically, the only viral transcripts thought to be expressed were the latency-associated transcripts (LATs), which are expressed in roughly 30% of HSV-1-infected neurons in vivo [33,71,72,73]. These transcripts are stable products of RNA splicing that are derived from transcription of the repeat regions neighboring the central unique region of the HSV-1 genome [74,75,76]. Functions encoded by the LAT locus lead to repression of lytic viral gene expression, thereby allowing for the establishment of latency in neurons [33,77,78,79,80,81]. Active repression of lytic viral gene expression provides for long-term infection as neuronal apoptosis is avoided [82]. Additionally, transmission can continue over the course of the patient’s lifetime during periods of reactivation [33,79,83]. Similar to the LATs, microRNAs (miRNAs) encoded within the LAT region also regulate lytic HSV-1 gene expression and play important roles in the establishment and maintenance of latency [84,85].

While it is true that the most abundant viral transcripts present during latency are the LATs and miRNAs, other data suggests that low levels of HSV-1 lytic gene expression may occur on a regular basis in specific populations of infected neurons. In the latently infected trigeminal ganglia (TG) of mice and rabbits, low-level expression of ICP4 and thymidine kinase is evident [86,87]. Within the TG of latently-infected mice, a small population of neurons (1 neuron per 10 TG) demonstrates lytic viral gene and protein expression, as well as viral DNA replication [88]. This occurs in the absence of infectious virion production and is characterized by the presence of immune cell infiltration [88,89]. This suggests that lytic viral gene expression is rapidly detected and addressed by the immune system. These data also indicate that rather than many latently-infected neurons within the TG expressing low levels of lytic viral genes, small populations express high levels [88], inducing prompt immune responses in the host’s attempt to maintain viral latency and avoid neuronal apoptosis.

Although lytic gene expression is observed, the presence of infectious virus in latently-infected ganglia is not [88]. Recent data suggests that reactivation from latency occurs in phases [90,91], which may also generate low levels of lytic gene expression. This may be an interesting parallel to the multiple stages of latency observed in Epstein–Barr virus infection [92,93]. Taken together, it can be hypothesized that HSV-1 causes a latent infection in neurons that “simmers” beneath the surface. This triggers persistent local parainflammation and immune responses to the low-level expression of lytic viral genes, which may aid in dampening viral gene expression and help maintain latency [94]. Parainflammation can be defined as a state in which inflammation is higher than basal, homeostatic levels, but less than a full inflammatory reaction [95]. One caveat to this assertion is that the studies referenced here were performed using mouse and rabbit in vivo models of HSV-1 latency, and HSV-1 is a highly human-specific virus in natural settings. However, these data are supported by evidence in human tissue demonstrating retrieval of HSV-1 from the trigeminal ganglia of cadavers, as well as the identification of lymphocyte infiltration therein [96].

## 3. Neuroinflammation, β Amyloid Plaques, and Neurodegeneration—Connections with Cognitive Decline and Alzheimer’s Disease

### 3.1. Expression of Immune Proteins in the Brain—Contributions of Neuroinflammation to Synaptic Dysfunction

HSV-1 establishes a latent, persistent viral infection that resides within neurons. What impacts does this type of infection have on the nervous system? What are the impacts of inflammation and inflammatory mediators in neural tissue? The concept of the nervous system as an immune-privileged site is derived from studies detailing the lack of cytotoxic T lymphocyte-mediated cell death in virus-infected neurons [97], as well as the inability of grafted tissue to be rejected when transplanted onto nervous tissue [98,99,100]. However, recent studies have detailed the contributions of immune proteins from both the innate and adaptive immune systems to neurodevelopment, normal brain homeostatic functions, neurodegeneration, and injury [61,62,63,64,65,66,67]. Specifically, proteins including the major histocompatibility class I (MHCI) protein, as well as the complement protein C1q and its receptor C3, are involved in synaptic refinement and neuronal connectivity [62,66,67]. Additionally, other inflammatory mediators are known to play critical roles in processes including learning and memory, neurodevelopment, and in the regulation of synapse strength [68]. These immune proteins and their receptors are expressed on neurons, glial cells, and microglia, which are the resident immune cells of the brain. Dysregulation of these neuroimmune pathways could underlie the premature synaptic loss that is associated with cognitive decline, neurodegeneration, and the development of neuropsychiatric disorders [67].

Aberrant expression of immune mediators in the brain is implicated in a number of neurological diseases [67,101]. This is particularly true in elderly populations as with increased age, there is an overall induction of inflammation. This has been termed “inflamm-aging”, a phenomenon describing the overall subclinical, chronic inflammatory status observed in elderly individuals [4]. For example, increased expression of neuroimmune proteins such as MHCI is observed with increased age in the brain [102,103,104]. MHCI expression in the brain is associated with the regulation of neurite outgrowth and synaptic connectivity during development [62,66]. However, aberrant expression of MHCI in the aged brain may trigger synapse loss. Synapse loss and atrophy in the absence of overt neurodegeneration is observed with advanced age [105,106], and premature synapse loss has been hypothesized to precede the onset of neurodegenerative disease and trigger cognitive decline [67]. Synaptic dysfunction is also associated with AD, often as a consequence of amyloid beta and tau pathology [26,107,108,109,110,111,112]. Together, these data indicate that overall inductions in the expression of inflammatory mediators with increased age may contribute to the manifestation of neurodegenerative disease including AD [18].

### 3.2. Pathological Hallmarks of AD

It is clear that inflammatory processes play a role in aberrant synaptic alterations over time, and potentially with the development of neurodegenerative disease. Could persistent infection with HSV-1 influence these neuroinflammatory processes and also contribute to AD pathology? The presentation of AD is initially diagnosed by a psychological evaluation indicating the manifestation of distinct changes in behavior and memory [8]. However, clinical diagnosis involves the identification of extracellular senile plaques, typically composed of amyloid beta and intracellular neurofilbrillary tau tangles, in areas of the brain critical for learning and memory [113]. Amyloid beta is composed of extracellular insoluble misfolded protein that aggregates and accumulates in the brain [114]. Accumulation of amyloid beta is present both in the brains of cognitively normal elderly individuals as well as in the brains of AD patients, suggesting that the presence of amyloid beta aggregates is not the sole cause of AD [113,115,116,117,118]. While the presence of amyloid beta peptides and/or plaques has been positively correlated with cognitive deficits [116,118,119], the relative contributions of amyloid beta plaques versus neurofibrillary tangles to dementia severity is a topic of debate [117]. Soluble amyloid beta is generated following cleavage of amyloid precursor protein (APP), a transmembrane protein involved in the formation of synapses, and in learning and memory [120,121]. Under normal conditions, soluble amyloid beta is hypothesized to be neurotrophic and participates in synaptogenesis and synaptic activity, neurodevelopment, learning and memory, and may have antioxidant properties [114]. Additionally, several research groups have indicated a role for amyloid beta in the restriction of pathogen movement and survival during microbial infection [21,22], making it an important factor in cellular immune responses, and providing a potential window for the involvement of HSV-1 in AD-associated neuropathology. However over-production of amyloid beta combined with decreased clearance of its aggregates may be neurotoxic and result in the formation of insoluble plaques [114]. As such, a delicate balance of amyloid beta production and clearance needs to be maintained, and it is this aspect which may be dysregulated with advanced age.

Another clinical indicator of AD is the presence of intraneuronal neurofibrillary tangles, which are composed of hyperphosphorylated tau [113]. Tau protein functions in microtubule assembly, where it binds to and stabilizes microtubules. When tau becomes phosphorylated, it does not bind microtubules as readily, resulting in microtubule disassembly and ultimately self-aggregation of tau [26,122]. Aberrant phosphorylation of tau results in distinct changes in neuronal cytoarchitecture [122,123,124], which may negatively impact synaptic stability and cognitive function. Precise regulation of tau phosphorylation is achieved via the actions of several different kinases and phosphatases [125,126]. These processes may become dysregulated with advanced age, resulting in increased accumulation of hyperphosphorylated tau, which is correlated with cognitive deficits observed in AD. Similar to amyloid beta, the presence of neurofibrillary degeneration is observed in specific areas in the brains of normal, non-demented elderly individuals as well as in AD patients, but not in younger individuals [127,128]. This suggests that neurofibrillary degeneration increases with age, however, there is no one-to-one relationship between the formation of neurofibrillary tangles and AD. As such, controversy exists surrounding the relative contributions of amyloid beta plaques and neurofibrillary tangles on the manifestation of cognitive deficits in AD, as well as whether these pathologies are true indicators of disease [117,129]. These data highlight that the etiology of sporadic AD is complex, and there is likely more than one mechanism that underlies disease pathology and cognitive deficits.

## 4. Possible Connections between Persistent HSV-1 Infection and AD

### 4.1. Persistent HSV-1 Infection and Neuroinflammation

**Figure 3:**
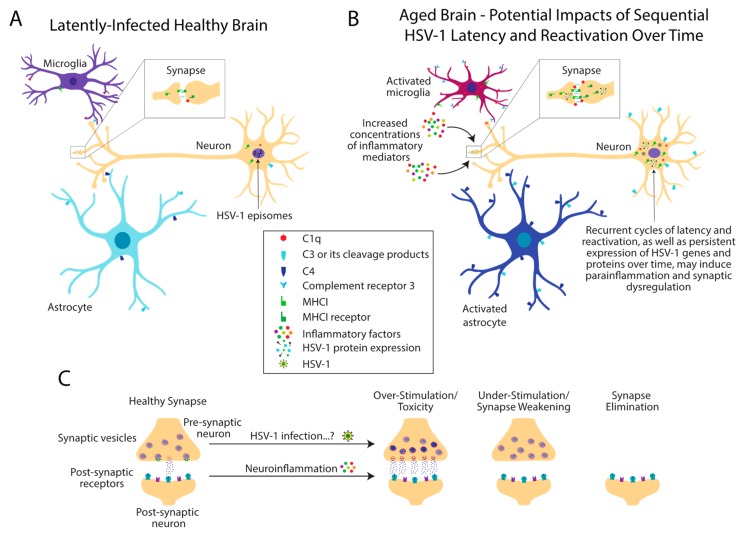
Recurrent cycles of HSV-1 latency and reactivation may cause aberrant neuroinflammatory responses and synaptic dysfunction with advanced age. (**A**) If HSV-1 enters the central nervous system (CNS), latency will be established in the nucleus of infected neurons. Neuronal and glial expression of immune factors including members of the complement pathway and the MHCI processing and presentation pathway are expressed at homeostatic levels. (**B**) Local immune responses triggered by the expression of lytic viral genes, combined with multiple cycles of HSV-1 reactivation from latency, may over time lead to the deleterious expression of immune proteins. Aberrant expression of these proteins may cause the activation of microglia and astrocytes, as well as the dysregulation of neuron-neuron and/or neuron-glial communication. (**C**) Altered expression of immune factors at neuronal synapses may lead to neuronal over-stimulation and toxicity, under-stimulation and synaptic weakening, and/or synaptic pruning initiated by microglia. Diagram: figure adapted from [136], cell type illustrations from BioRender.com.

The ability of HSV-1 to maintain a persistent infection within neurons allows the virus to evade immune clearance, resulting in lifelong infection. Although the benefits of neuronal infection for HSV-1 pathogenesis and transmission are quite clear, the long-term consequences of infection on host neurons later in life have yet to be fully elucidated. Repeated cycles of HSV-1 latency and reactivation over time may cause detrimental effects to infected neurons as well as the surrounding microenvironment, leading to subsequent disease (Figure 3) [24]. This is true not only in the nervous system, but also in other niches throughout the body. For example, HSV-1 infection is linked to the development of coronary heart disease and myocardial infarction, and has been identified within atherosclerotic lesions [130,131,132]. It is possible that a history of frequent HSV reactivation concurrent with increased anti-HSV antibody levels [133,134] may increase the risk for lethal myocardial infarction and coronary heart disease [134,135]. 

Recent data has implicated persistent infection with HSV-1 to the development of AD. The main site of HSV-1 latency is in the peripheral nervous system (PNS), and it is thought to only rarely enter the CNS. So how then can peripheral HSV-1 infection trigger neurodegenerative processes in the CNS? One of two hypotheses may be possible. The first hypothesis is that chronic peripheral inflammation, combined with increases in blood-brain-barrier permeability with advanced age, may lead to higher immune cell and inflammatory mediator infiltration into the CNS, resulting in aberrant neuroinflammation and neurodegeneration. The second hypothesis is that HSV-1 may travel via synaptically connected neurons beyond the peripheral ganglia, and maintain latent subclinical infection within the CNS, causing local neuroinflammation and neurodegeneration in regions of the brain often affected by AD. Evidence discussed below exists to support both of these hypotheses, suggesting that these are not mutually exclusive ideas.

With advanced age, there is an increase in blood-brain-barrier permeability, as well as immunosenescence, a term used to describe the gradual dysfunction of the immune system and overall reduction of immune responses to different perturbations [137,138]. With the onset of immunosenescence, it is possible that immune control of HSV-1 latency in peripheral ganglia may diminish, similar to what is seen with other herpesviruses, making infection more chronic, rather than latent, at later stages in life [139]. Increased cycling between latency and reactivation may induce enhanced expression of inflammatory factors, such as cytokines, that are targeted at dampening lytic HSV-1 gene expression in infected neurons. Over time, focal immune cell infiltration in the peripheral ganglia may increase. If this were to occur simultaneously with age-associated increases in blood-brain-barrier permeability [137], this could potentially lead to higher than normal levels of immune cell and inflammatory mediator infiltration into the brain. Even if increased cycling between latency and reactivation did not occur with increasing age, chronic local parainflammation in the peripheral ganglia that is targeted at maintaining HSV-1 latency, combined with increased blood-brain-barrier permeability, could lead to the same outcome of enhanced immune infiltration into the brain.

If there are frequent, low-levels of inflammation in the nervous system as a result of immune-control of latent HSV-1 infection [88,89,94], then local alterations in synapse function and aberrant synaptic pruning may also occur (Figure 3). This could result in the development of cognitive impairment later in life, and perhaps ultimately AD. However, it is likely that latent infection of HSV-1 in the peripheral ganglia is not the only factor contributing to enhanced peripheral and/or central inflammation. For example, expression of the immune mediator IL-6 is dysregulated with advanced age [140]. This results in enhanced circulating serum IL-6 concentrations, which is hypothesized to play detrimental roles in the progression of many different age-associated complications [140]. As such, persistent peripheral HSV-1 infection is more likely to be a contributing factor, rather than a sole trigger, of the process of inflamm-aging [4].

There is also the possibility that HSV-1 may enter neurons of the CNS over the course of a patient’s lifetime, during one of the many cycles of latency and reactivation. This could potentially underlie the introduction of focal areas of inflammation into specific areas of the CNS known to be affected in AD. HSV-1 has been identified in the brains of immunosuppressed individuals [141]. There is also evidence of HSV-1 in the brains of AD patients as well as in healthy elderly brains, suggesting that central nervous system infection with HSV-1 is not the only risk factor for AD development [142,143,144,145,146,147]. Importantly, samples derived from the brains of younger individuals do not have detectable levels of HSV-1 [145]. Together, these data suggest that increased penetrance of HSV-1 into the brain occurs with advanced age. It has been hypothesized that HSV-1 can travel to regions of the brain that are important for learning and memory, as areas such as the frontal and temporal lobes are preferentially affected during acute HSV-1-induced encephalitis [148,149]. Increased penetrance of HSV-1 into the brain with advanced age may contribute to the development of AD pathology in these regions. In support of this hypothesis, HSV-1 DNA has been identified within amyloid plaques in the brains of AD patients [150].

The data discussed here provide exciting insights, suggesting that age and HSV-1 infection are associated with AD development. Understanding the relationship between HSV-1 infection and AD pathogenesis may allow for the development or increased usage of anti-viral therapeutics that may aid in either preventing or allaying AD development. However, conflicting data exists that have demonstrated relatively few AD and healthy aged brains containing HSV-1 [151,152]. These latter data, combined with other studies demonstrating the presence of HSV-1 in the healthy aged brain [145,146], highlight the fact that HSV-1 infection cannot be the sole factor underlying the development of sporadic AD. Rather, complex virus–host interactions are likely occurring that contribute to AD pathogenesis, including ones that may be particular to individual virus strains, human hosts, and environmental factors (Figure 1).

### 4.2. Implications for HSV-1 Infection in the Development of AD Pathology

The potential molecular mechanisms whereby HSV-1 infection may contribute to AD pathology have been recently reviewed in a comprehensive manner [26]. Neuronal infection with HSV-1 has been linked to amyloid beta accumulation, hyperphosphorylation of tau, neuroinflammation, and synaptic dysfunction [26]. Increased amyloid beta expression and accumulation following infection with HSV-1 have been demonstrated in vitro and in vivo, respectively [26,34,153]. This induction in amyloid beta deposition may be the result of an upregulation in the expression of key amyloid beta processing components in response to infection [153]. In adult hippocampal neural stem cells, amyloid beta protein accumulates in response to HSV-1 infection, leading to decreased proliferation and differentiation both in vitro and in vivo [154]. These data have important implications for the impacts of recurrent cycles of latency and reactivation on learning and memory loss with age and AD development. Increased expression of the tau phosphorylating enzymes GSK3β and PKA following HSV-1 infection may trigger the tau hyperphosphorylation observed in HSV-1-infected neurons in vitro [26,155,156,157]. In addition to an upregulation in neuroinflammation [158,159,160], HSV-1 triggers synaptic dysfunction and synapse loss via activation of GSK3β and amyloid beta accumulation [38]. Alterations in synaptic stability and function may also be an indirect result of the accumulation of hyperphosphorylated tau and aberrant cytoskeletal rearrangement, as well as an off-target consequence of neuroinflammation.

All of the above molecular consequences of HSV-1 infection have also been implicated in the manifestation of cognitive deficits associated with AD. In addition, neuronal infection with HSV-1 triggers a number of different transcriptional changes in host neurons, including alterations in immune response, cell cytoarchitecture, extracellular matrix, and neuronal metabolism [161]. Over time, these changes in neuronal physiology may adversely affect neuron-neuron and/or neuron-glia signaling, resulting in synaptic dysfunction and aberrant synaptic pruning. Together, these studies link HSV-1 infection with the development of AD pathology in infected neurons.

## 5. Strain-Specific Differences in HSV-1 Neurovirulence and the Potential Impact on AD Development

Recent studies have demonstrated that extensive genetic diversity exists between circulating HSV-1 strains, as well as within the pool of virus (i.e. virions or viral genomes) associated with any one infection [47,48,162]. Other studies have highlighted the impact of specific polymorphisms on differences in pathogenesis and neurovirulence observed between different strains of HSV-1 [163,164]. The term neurovirulence is used to describe a virus’s ability to directly impact the nervous system and cause disease [165]. Neurovirulence is the sum total of multiple underlying components, which include the ability of a given virus strain to invade the nervous system (neuroinvasion), its cell-to-cell spread capabilities, the ability to replicate in neurons and cause overt disease, and its ability to reactivate from latency [49,166]. The correlation of specific genetic differences between HSV-1 strains to phenotypic outcomes in viral fitness and neurovirulence is therefore of significant clinical relevance. In different strains of HSV-1, the comparison of viral genotype to disease phenotype has revealed connections between ocular disease and specific viral protein variants [167]. These data suggest that polymorphisms in specific viral proteins may impact cell-to-cell spread and neurovirulence. Likewise, a recent comparison of fraternal (dizygotic) vs. identical (monozygotic) human twins with HSV-1 infection provided evidence suggesting that differences in oral shedding rate were linked to viral genetic factors, rather than to human differences [59]. Elucidating the contributions of these variants to HSV-1 pathogenesis has significant implications, not only for understanding the mechanisms of acute disease, but also to understanding the complications of persistent infection to disease occurrence later in life.

**Figure 4:**
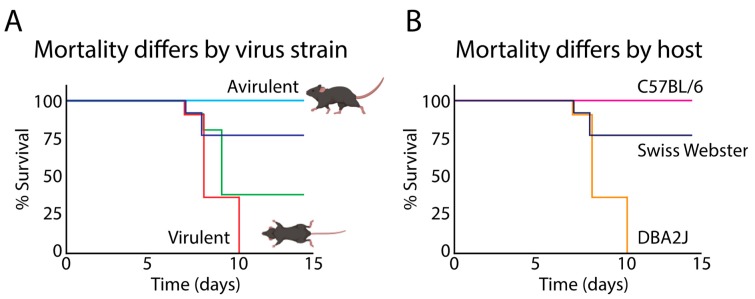
HSV-1 neurovirulence is strain- and host-dependent. (**A**) Different strains of HSV-1 (as represented by the different colored lines) exhibit distinct abilities to reach the CNS from inoculation at peripheral sites, causing variability in disease outcome in infected animals. Differences in the ability of specific HSV-1 strains to travel to the CNS can be the result of genomic variability between strains as well as (**B**) host-specific factors. These are schematized mortality data adapted from [49,52]. Mouse illustrations from BioRender.com.

In animal models of infection, different strains of HSV-1 possess varying abilities to reach the CNS after inoculation at peripheral sites (Figure 4), suggesting that there are strain-specific differences in neuroinvasion, which is a critical component of neurovirulence [49,50,168]. Importantly, this appears to reflect differences in neuron-to-neuron spread capabilities between strains rather than differences in their ability to infect and replicate in neurons. For instance, coding variations in virion surface glycoproteins have been detected between strains, which have been shown to influence viral entry and intercellular spread [58,169,170]. Other essential components of an HSV-1 strain’s neurovirulence include its ability to cause overt disease and to reactivate from latency [166]. In rabbit ocular models of HSV-1 infection, inoculation with a commonly used laboratory strain, McKrae, yielded roughly 50% mortality, and a high rate of spontaneous reactivation following latency. In contrast, infection with a different strain, KOS, yielded no mortality and low rates of spontaneous reactivation [83,168,171]. These differences in disease severity and ability to spontaneously reactivate may in part be due to distinct strain-specific polymorphisms within key viral genes such as ICP34.5 and LAT [170,171]. As mentioned above, the LATs play key roles in the repression of lytic viral gene expression, as well in controlling reactivation [33,77,78,79,80,81]. Differences in LAT expression or function between strains may impact the virus’s ability to enter and/or maintain latency, resulting in disparities in disease pathogenesis. The viral gene ICP34.5 exhibits variation between HSV-1 strains. ICP34.5 encodes a neurovirulence factor that controls viral protein expression and replication in the nervous system [172,173], as well as autophagy in infected neurons [174]. Importantly, ICP34.5 deletion and truncation mutants demonstrate altered neurovirulence in animal models, as does insertion of ICP34.5 from an avirulent strain into a neurovirulent strain backbone [171,172,174,175]. Additionally, it has been hypothesized that variations in ICP34.5 coding sequences results in alternative protein localization between strains, and may influence neurovirulence [176]. Together, these data highlight that polymorphisms within specific viral genes may play important roles in regulating the neurovirulence of a given HSV-1 strain. These genetic differences between virus strains may ultimately have differential impacts on the contributions of persistent HSV-1 infection to the development of AD. Yet to date, most studies of HSV-1 and AD molecular interactions have examined only one strain at a time [24,153,155,157,177,178,179,180,181,182,183]. Future research should therefore seek to understand the phenotypic consequences of distinct strain polymorphisms on neurovirulence traits, as well as strain-specific impacts on host neuronal physiology and potential AD-related neuropathology over time.

It is important to note that host-specific factors also play key roles in the regulation of HSV-1 neurovirulence. Not all individuals who are infected with HSV-1 develop AD, nor do all patients with evidence of CNS dissemination of HSV-1 develop AD [144]. There may be both virus- and host-specific factors that trigger differences in host immune responses and/or susceptibility to virus-induced neuronal damage. For instance, viral strain differences in replication or reactivation rate may manifest as differences in lesion intensity of frequency of reactivation. In addition, differences in human immune detection (intrinsic cell defenses) and/or adaptive immune responses may lead to variations in the efficacy of immune control and thus the duration of periods of latency. HSV-1-encoded glycoproteins, gE and gI, together form a heterodimer that functions as an Fc gamma receptor (FcγR) and binds to host immunoglobulin (IgG), contributing to immune evasion [184,185]. The HSV-1 FcγR demonstrates specificity in binding to different IgG1 allotypes [186], suggesting that variability may exist between individuals in their ability to mount effective immune responses against both lytic and latent HSV-1 infection [187]. Over time, virus strain- and host-specific interactions such as these may cause different degrees of parainflammation between patients, resulting in differential impacts on the probability of AD development later in life. In support of this hypothesis, associations between mild cognitive impairment, AD, and anti-HSV-1 antibody levels were recently found to vary dependent on immunoglobulin gamma marker genotype [188].

Genetic differences in anti-HSV-1 immune responses, compounded by infection with more neurovirulent strains of HSV-1, may exacerbate the development of cognitive deficits with age. Additionally, infection with a highly neurovirulent strain of HSV-1 combined with a genetic predisposition for AD development may also increase an individual’s’ risk, as previously hypothesized [189]. This is supported by evidence identifying specific host genetic loci that influence HSV-1 disease pathogenesis [52]. One specific example is carriage of the apolipoprotein E ε4 (APOE4) allele, a known genetic risk factor for the development of AD [190,191]. Recent data has shown a positive association between HSV-1 infection, carriage of the APOE4 allele, and declining episodic memory function in elderly individuals [192]. The apoE4 isoform has been hypothesized to facilitate HSV-1 entry and latency in the brain more readily than other apolipoprotein isoforms [193,194]. Relative neurovirulence of any particular HSV-1 strain may therefore be compounded with a host’s genetic predisposition for AD, such as carriage of the APOE4 allele, resulting in more rapid and/or more advanced disease development. However, further analyses need to be done to assess the relationship between HSV-1 strain and APOE4 allele variation.

The connection between neuronal HSV-1 infection and the accumulation of amyloid beta and hyperphosphorylated tau are clear [see references above and reviewed in [26]]. To date, no studies have been performed examining whether there are virus strain-specific differences in the generation and accumulation of these neurotoxic proteins, and how variation between individual hosts also contributes to these pathologies. Future studies should aim to identify and investigate how variations between specific hosts and virus strains lead to differences in observed HSV-1 neurovirulence, how these impact neuronal physiology over time, and the contributions of these factors to disease development. Targeted antiviral therapies can then be used in a way that matches the specific therapeutic approach to the precise pathogen strain present and the host’s own ability to control that virus strain. In this way, the development of AD pathology and cognitive dysfunction can be potentially slowed or inhibited.

## 6. Conclusions

The etiology of sporadic AD is complex, and is hypothesized to involve both environmental and genetic factors. It is becoming increasingly clear that persistent infection by microbes such as HSV-1 may play important roles in the development of sporadic AD. Our understanding of the association between neuronal infection with HSV-1 and AD is just beginning to surface. Repeated cycles of HSV-1 latency and reactivation may trigger chronic central and/or peripheral parainflammation, which over time causes synapse loss, potentially leading to the cognitive deficits associated with AD. Additionally, neuronal infection with HSV-1 may contribute to the accumulation of amyloid beta deposits and hyperphosphorylated tau that are associated with AD pathology. Together, these characteristics of HSV-1 infection may increase the risk of developing sporadic AD. More research is needed to definitively understand the molecular mechanisms of how virus strain- and host-specific factors intersect to impact disease pathology and development. Understanding the specificity and individualized nature of these lifelong host-virus interactions may lead to the identification of novel therapeutic opportunities to prevent or slow the progression of AD.
